# Thorium Valorization at the Interface of Technology, Risk, and Sustainability

**DOI:** 10.3390/toxics14030193

**Published:** 2026-02-25

**Authors:** Geani Teodor Man, Andreea Maria Iordache, Diana Ionela Popescu (Stegarus), Ionela Ramona Zgavarogea, Nicoleta Anca Șuțan

**Affiliations:** 1 National Research and Development Institute for Cryogenics and Isotopic Technologies—ICSI Râmnicu Valcea, 4th Uzinei Street, 240050 Ramnicu Valcea, Romania; geani.man@icsi.ro (G.T.M.); andreea.iordache@icsi.ro (A.M.I.); diana.stegarus@icsi.ro (D.I.P.); 2 Department of Analytical Chemistry and Environmental Engineering, National University of Science and Technology POLITEHNICA Bucharest, Bucharest University Centre, Splaiul Independenţei nr. 313, Sector 6, 060042 Bucharest, Romania; 3 Department of Natural Sciences, National University of Science and Technology POLITEHNICA Bucharest, Piteşti University Centre, Targul din Vale 1, 110040 Pitesti, Romania

**Keywords:** thorium, rare earth elements, extraction technologies, environmental mobility, toxicological risk, circular economy, sustainable management

## Abstract

Thorium (Th), a naturally occurring actinide, is gaining renewed attention due to its dual role as a strategic nuclear resource and a potential environmental contaminant. This review critically reassesses thorium valorization pathways by integrating extraction technologies, environmental behavior, toxicological risks, and regulatory constraints. While thorium is primarily recovered as a by-product of rare earth element (REE) processing, conventional hydrometallurgical methods—though mature—generate significant secondary waste and pose environmental challenges. Emerging technologies, such as functionalized adsorbents, membrane systems, and biohydrometallurgy, show promise but remain largely confined to laboratory-scale studies due to scalability and stability issues. A key finding is that thorium’s environmental mobility and toxicological impact are directly influenced by the extraction processes used, creating species with distinct bioavailability and risk profiles. This work highlights the disconnect between high laboratory efficiencies and real-world applicability, emphasizing the need for integrated approaches that consider lifecycle impacts, waste minimization, and occupational safety. We propose a circular economy framework for sustainable thorium management, connecting green primary processing, secondary recovery from industrial residues, smart environmental stewardship, and supportive policy. The review concludes that successful thorium valorization depends not on incremental efficiency gains but on holistic designs that reconcile technological performance with environmental and health safeguards.

## 1. Introduction

Thorium (Th) is a naturally occurring actinide widely distributed in the Earth’s crust, with an average abundance of approximately 9–10 ppm in the bulk continental crust in specific igneous and metasedimentary formations; concentrations may increase to several tens or even hundreds of grams per ton (ppm) [1,2,3,4,5,6].

Due to its comparable ionic radius to U^4+^ under similar coordination conditions and its tetravalent oxidation state, thorium commonly occurs alongside uranium, particularly in alkaline magmatic systems and rare earth element (REE)-rich pegmatites, where both elements substitute for high-field-strength cations in accessory minerals [7,8]. It occurs predominantly as the long-lived isotope ^232^Th, which accounts for nearly all natural thorium; although ^232^Th has a very long half-life, the main radiological concern is associated with its daughter radionuclides formed along the decay chain [9].

Interest in thorium has intensified over the past two decades due to its dual status as both a strategic resource and a potential environmental contaminant. On one hand, thorium has been promoted as a fertile nuclear material capable of breeding fissile ^233^U, offering theoretical advantages over conventional uranium-based fuel cycles in terms of resource abundance, fuel utilization efficiency, and reduced production of long-lived transuranic waste [10,11]. This renewed interest is inextricably linked to the rapid expansion of the REEs sector, as thorium is predominantly recovered as a by-product of REE mining and processing. The strategic importance of REEs for modern energy technologies has consequently reopened discussions on thorium valorization within the framework of sustainable nuclear energy systems. Consequently, life cycle assessment (LCA) approaches have been increasingly applied to evaluate the environmental, radiological, and socio-economic indicators of thorium fuel cycles [12]. On the other hand, thorium is classified as a radioactive heavy metal with recognized toxicological risks, particularly when mobilized through mining, processing, or waste disposal activities [13]. This intrinsic duality places thorium at the intersection of energy policy, environmental protection, and public health.

Beyond nuclear energy, thorium has been utilized in a range of industrial and technological applications, including high-temperature ceramics, catalysts, optical components, and historically in gas mantles and radiological contrast agents [14,15,16]. While these applications highlight thorium’s technological value, they also amplify concerns related to occupational exposure, waste generation, and long-term environmental contamination [17,18,19].

The extraction and separation of thorium are rarely conducted as stand-alone processes. Instead, thorium is predominantly recovered as a by-product during REE mining and beneficiation, where its presence is often undesirable due to radiological constraints and regulatory limits [20]. As a result, a wide spectrum of chemical, physical, and biological separation techniques has been proposed, ranging from conventional acid or alkaline leaching and solvent extraction to advanced approaches such as membrane technologies, adsorption on functionalized materials, and biohydrometallurgical processes [21,22,23,24]. However, despite the extensive volume of published research, the majority of these methods remain confined to laboratory or pilot-scale studies, with limited evidence of industrial viability.

At the same time, thorium released into the environment through mining tailings, fertilizer production, coal combustion, and nuclear-related activities exhibits low solubility but strong affinity for particulates, sediments, and biological interfaces [25,26,27]. These properties govern its environmental fate, transport, and bioavailability, ultimately influencing exposure pathways for humans and non-human biota. Epidemiological and experimental studies have linked thorium exposure, particularly via inhalation and ingestion, to increased risks of lung cancer, liver dysfunction, genotoxicity, and other chronic health effects, largely driven by alpha radiation emitted during radioactive decay [17,18,19,25]. Nevertheless, available toxicological data are often fragmented, derived from heterogeneous exposure scenarios, or extrapolated from uranium-focused studies, limiting robust risk assessment.

Despite the breadth of existing literature, several critical issues remain insufficiently addressed. First, there is no consensus on which thorium separation and recovery technologies are realistically scalable when energy consumption, secondary waste generation, and regulatory constraints are taken into account. Methods frequently described as “highly efficient” in laboratory settings often rely on large volumes of hazardous reagents, expensive functional materials, or complex multi-step processes that undermine their industrial applicability [28,29]. Second, the relationship between thorium valorization and environmental or occupational risk is rarely analyzed in an integrated manner. Extraction efficiency is typically discussed independently of downstream waste management, exposure scenarios, and long-term health implications [17,30].

In this context, the objective of this review is to critically reassess thorium valorization pathways by jointly examining extraction and separation technologies, environmental behavior, and toxicological risks. Rather than providing an exhaustive catalogue of available methods, this work focuses on identifying technological bottlenecks, contradictions within the literature, and gaps between experimental performance and real-world applicability. By integrating technological, environmental, and health-related perspectives, this review seeks to clarify which approaches hold genuine potential for sustainable thorium management and which remain primarily academic constructs. Such an assessment is particularly timely given the renewed global interest in thorium-based energy systems and the expanding scale of rare earth mining, both of which are likely to intensify thorium-related environmental and public health challenges in the near future.

## 2. Sources and Occurrence of Thorium in Natural and Technological Systems

Thorium is commonly associated with REE minerals such as monazite, bastnäsite, xenotime, perovskite–loparite, pyrochlore, britholite, and, to a lesser extent, zircon, as well as in ion-adsorption clays. It is frequently present in technological residues generated during REE extraction and processing, where it represents both a regulatory burden and a potential secondary resource [21,31,32,33]. Thorium resources occur mainly in placer, carbonatite, and vein-type deposits and are hosted by minerals such as monazite, thorite, and thorianite. According to the OECD Nuclear Energy Agency, globally identified thorium resources are estimated at about 6.4 million tons, with the largest shares in India, followed by Brazil, Australia, and the United States [34].

Thorium is a naturally occurring actinide widely distributed in the Earth’s crust, with an average abundance higher than that of uranium, although its spatial distribution is highly heterogeneous [9,35,36]. From a geochemical perspective, thorium is only tetravalent (Th^4+^) and exhibits strong lithophile behavior, resulting in its preferential incorporation into accessory minerals rather than major rock-forming phases [37,38,39]. While this general behavior is well established, its practical relevance lies less in mineralogical classification and more in the technological and regulatory consequences of thorium’s co-occurrence with economically valuable resources [21,40].

A defining characteristic of thorium occurrence is its close association with REE-bearing minerals, particularly monazite, bastnäsite, xenotime, and related phases [38,41,42]. In these systems, thorium is rarely present as an independent target element. Instead, it accompanies REEs as a chemically persistent and radiologically regulated component. This association has direct implications for extraction strategies, as thorium recovery is often driven not by economic demand but by the necessity to manage radioactive content during REE processing. Consequently, thorium is frequently treated as an unwanted by-product, influencing process flowsheets, waste classification, and downstream handling requirements [43,44].

Beyond primary mineral deposits, thorium is increasingly encountered in technological and industrial residues. Significant amounts have been reported in tailings from REE mining and beneficiation [45,46,47,48], phosphogypsum generated during phosphate fertilizer production [49], coal combustion residues [50], and red mud derived from alumina processing [32,51]. In these secondary matrices, thorium concentrations are generally lower than in primary ores but are often more environmentally relevant due to increased surface area, altered chemical speciation, and greater potential for dispersion. From a risk perspective, these residues represent diffuse sources of thorium release rather than controlled extraction points [27,52,53].

The technological relevance of thorium occurrence in such residues is related to both potential resource valorization and environmental management considerations. On one hand, secondary materials have been proposed as alternative thorium sources, potentially reducing the need for new mining activities [54]. On the other hand, the heterogeneous composition and variable physicochemical properties of industrial wastes complicate separation processes and raise concerns regarding secondary waste generation and occupational exposure [55,56].

Importantly, the form in which thorium occurs in natural and technological systems strongly influences both its processability and environmental behavior. Thorium bound within resistant mineral lattices exhibits limited mobility, whereas thorium associated with fine particulates, amorphous phases, or process-derived compounds may display enhanced reactivity under changing physicochemical conditions [25,57,58,59,60]. This distinction should be considered when evaluating separation strategies, as aggressive chemical treatments designed to liberate thorium from solid matrices may simultaneously increase its environmental availability and radiological risk [44].

Rather than serving as a purely descriptive background, the sources and occurrence of thorium therefore provide contextual information relevant to the development of valorization or remediation strategy [61]. The persistent coupling of thorium with REE production chains, combined with its presence in diverse industrial residues, ensures that thorium management remains as much a regulatory and environmental challenge as a technological one [16,62,63]. Knowledge of thorium occurrence can support the optimization of separation processes and help anticipate potential implications of thorium mobilization across natural and engineered systems.

Materials containing elevated concentrations of naturally occurring radioactive materials (NORM) are subject to specific control regimes under the International Atomic Energy Agency Safety Standards, which establish dose limits, exemption criteria, and requirements for occupational and environmental monitoring. Within the European Union, the Euratom Basic Safety Standards Directive (2013/59/Euratom) integrates NORM industries, including rare earth processing and mineral sands operations, into a harmonized radiological protection framework based on justification, optimization, and dose limitation principles.

These regulatory instruments influence technology deployment by imposing authorization procedures, radiological characterization of residues, long-term waste management obligations, and worker dose monitoring. At the same time, they may enable controlled valorization pathways when exposure scenarios remain below regulatory thresholds and when stabilization measures are demonstrably effective. Consequently, the feasibility of thorium recovery from both primary and secondary resources is closely linked to compliance costs, waste classification criteria, and long-term stewardship requirements defined by international and regional radiation protection systems [13,64,65,66,67].

## 3. Thorium Extraction and Separation Technologies: A Critical Assessment

The extraction and separation of thorium have been investigated for decades, largely in connection with REE processing [68] and nuclear fuel cycle research [36,69]. While a broad range of techniques has been proposed, their technological maturity, environmental footprint, and industrial relevance vary substantially. Rather than providing an exhaustive catalogue of available methods, this section compares the main technological approaches based on common criteria, including chemical efficiency, process robustness, waste generation, and scalability.

### 3.1. Conventional Hydrometallurgical Approaches

Conventional hydrometallurgical methods are among the well-established strategies reported in the literature and widely applied for thorium recovery, primarily due to their compatibility with established REE processing flowsheets. Acidic leaching, typically using sulfuric acid (H_2_SO_4_), nitric acid (HNO_3_), or hydrochloric acid (HCl), but also condensed phosphoric acid [70] is the dominant approach for mobilizing thorium from monazite [71], bastnäsite [72], xenotime [73] and related matrices, while alkaline digestion with sodium hydroxide [74] and more recently with potassium hydroxide [75] has been applied in selected cases to improve selectivity or reduce silica dissolution [76,77].

From a technical standpoint, these methods can achieve high thorium dissolution efficiencies under controlled conditions. However, such performance is often associated with aggressive chemical environments, elevated temperatures, and low solid-to-liquid ratios, all of which impose significant constraints on industrial implementation [58,78]. Moreover, acidic leaching processes frequently solubilize multiple co-existing elements, necessitating complex downstream separation steps and increasing the volume of secondary radioactive waste [68,79].

Solvent extraction represents the most established technique for thorium separation from leach liquors, employing extractants such as organophosphorus compounds, tributyl phosphate (TBP), Di(1-methyl-heptyl) methyl phosphonate (DMHMP) [80], D2EHPA [81], and Cyanex-type reagents [82]. These systems offer high selectivity when process parameters are carefully optimized, and they have been successfully integrated into pilot-scale operations [74,75]. Moreover, Wang et al. [83] reported that a synergistic mixture of Cextrant 230 and Cyanex 923 improved Th^4+^ extraction performance in chloride media, with increased thorium loading, facilitated stripping, and reduced rare earth co-extraction compared to single extractants.

Nevertheless, solvent extraction circuits are chemically intensive, sensitive to feed composition, and generate spent organic phases and acidic raffinate streams that require careful management.

Overall, while conventional hydrometallurgical approaches provide established routes for thorium recovery, their efficiency could be evaluated alongside reagent consumption, waste neutralization requirements, and long-term environmental liabilities, especially under stricter regulatory and sustainability frameworks [40,61].

### 3.2. Advanced and Emerging Technologies

In response to the limitations of conventional methods, a wide range of advanced and emerging separation technologies has been proposed, often with the aim of improving selectivity, reducing chemical consumption, or enabling thorium recovery from low-grade or secondary resources [22,84,85,86,87,88,89,90,91,92]. Among these, functionalized adsorbents [24,93,94,95], membrane-based systems [92,96,97], and biohydrometallurgical approaches [98,99,100,101] have attracted particular attention.

Solvometallurgy is a low-temperature extractive approach that can be applied to metals including thorium, particularly from complex ores, tailings, and secondary resources where conventional aqueous processing is inefficient. By replacing a discrete aqueous phase with low-water, non-aqueous solvents, solvometallurgy enables more selective leaching and separation of metal ions, a feature directly relevant to thorium chemistry given its strong solvation and coordination behavior in organic media. The integration of solvent leaching and separation into a single step reduces water consumption, avoids wastewater generation, and minimizes acid use, providing potential benefits for environmentally safer thorium processing compared with classical hydrometallurgy. Although the technology readiness remains low (TRL 3–4), solvometallurgy has been investigated as a research direction for thorium extraction that may minimize waste when green, low-toxicity, and recyclable solvents are employed [102].

Functionalized solid adsorbents, including ion-exchange resins [103], metal–organic frameworks [104,105,106,107], graphene-based materials [78,90,108], and modified polymers [97,109], frequently demonstrate high thorium affinity and selectivity under laboratory conditions. These materials are often presented as environmentally benign alternatives to solvent extraction. However, their performance is typically evaluated at low thorium concentrations, narrow pH ranges, and small solution volumes, limiting the relevance of reported capacities to real industrial streams. In addition, challenges related to adsorbent synthesis, regeneration efficiency, and long-term stability remain insufficiently addressed.

Electrosorption has been investigated as a strategy for selective thorium recovery, addressing the limitations of conventional adsorption by using an electric field to accelerate ion migration and enhance adsorption rates. Functionalized electrodes, such as amidoxime-modified graphite felt (GF-AO), g-C3N4, activated carbon electrodes (ACE), and graphene-based electrodes (GBE), demonstrate high thorium affinity and selectivity even in the presence of competing rare earth elements [78,91,110]. Many advanced materials are optimized against idealized Th^4+^ species that rarely dominate real leachates, which can limit their practical performance under complex industrial conditions. The GF-AO electrode achieves exceptionally high electrochemical recovery capacities (>3000 mg·g^−1^) and rapid sorption–desorption cycles [91], while g-C3N4 electrodes show a threefold increase in capacity under applied voltage, driven by synergistic electrostatic and chemisorption interactions [110]. Comparative studies indicate that graphene-based electrodes outperform activated carbon electrodes in both thorium recovery efficiency (~92%) and environmental performance, with lower human toxicity, freshwater and marine ecotoxicity, and resource impacts, highlighting the importance of electrode material choice for sustainable radioactive waste management [78]. Both GF-AO and g-C3N4 electrodes exhibit good reusability and stability, reduce chemical consumption, and offer potential for integration with renewable energy sources, underscoring electrosorption as a low-carbon, efficient, and potentially industrially relevant method for thorium recovery from aqueous solutions and complex residues [91,110].

Conceptually, electrosorption provides enhanced control over ion selectivity and overcomes diffusion limitations through electric-field-assisted adsorption [85,111]. In practice, however, its effectiveness is limited by the availability and long-term stability of high-capacitance functional electrodes, low throughput, and sensitivity to competing ions, which constrain its scalability beyond laboratory demonstrations [110]. Consequently, the technology readiness level remains low to moderate, reflecting strong potential but significant challenges for industrial deployment.

Membrane-based separation techniques, such as supported liquid membranes [112,113] and polymer inclusion membranes [114], offer the theoretical advantage of combining extraction and stripping in a single step. Although some recent studies reported significant improvement in selectivity, permeability and fouling resistance [115], membrane systems are inherently sensitive to fouling, mechanical degradation, and fluctuations in feed composition. These factors, coupled with limited throughput, currently restrict their application to proof-of-concept or niche scenarios.

Bioleaching and biosorption approaches have also been explored as low-energy alternatives for thorium mobilization, particularly from low-grade ores and industrial residues [116,117,118]. Although microbial activity and biomass-based sorbents can interact with thorium-bearing phases, process kinetics are generally slow, microorganisms could be inhibited by toxic metals and the reproducibility of results across different matrices remains problematic [119,120,121].

Taken together, advanced separation technologies provide information on alternative thorium binding mechanisms but remain largely constrained to laboratory-scale demonstrations. Their transition to industrial application is hindered by material complexity, process sensitivity, and insufficient evaluation under realistic operating conditions.

To move beyond descriptive comparisons, the main thorium extraction and separation approaches reported in the literature are summarized comparatively in Table 1. Taken together, advanced separation technologies provide innovative insights into thorium recovery mechanisms and environmental risk reduction, but most remain confined to laboratory or pilot-scale demonstration due to material complexity, process sensitivity, and insufficient evaluation under realistic operating conditions.

### 3.3. Scalability and Waste Challenges in Thorium Extraction

A persistent issue in developing thorium separation methods, from conventional solvent extraction to novel adsorbents, is the absence of a standardized method for evaluating scalability, waste management, and regulatory considerations in industrial deployment [29,61]. High extraction yields in a controlled laboratory setting may not directly correspond to industrial feasibility due to challenges of reagent recycling, effluent detoxification, and ensuring worker safety against radiation exposure [85].

A common shortfall is the narrow focus on chemical efficiency at the expense of the total waste footprint. For instance, while advanced MOFs boast exceptional selectivity for thorium in pure solutions, their practical application is hindered by complex synthesis, poor stability in acidic industrial liquors, and the unresolved cost of regenerating or disposing of the spent material [93,106]. The resulting secondary streams, whether spent solvents, loaded adsorbents, or acidic sludges containing residual radioactivity, represent not just a technical problem but the primary driver of operational cost and regulatory complexity, particularly under strict controls for naturally occurring radioactive materials (NORM) [105,124].

Furthermore, the leap from a beaker to a continuous plant is rarely tackled in earnest. Critical engineering parameters, such as the handling of solid feed variability, the long-term stability of phase separation in mixer-settlers, or membrane fouling in real wastewater, are frequently overlooked in favor of optimizing batch performance [85]. Limitations in assessing process robustness under non-ideal conditions may contribute to discrepancies between laboratory results and plant-scale feasibility.

Activated carbon obtained from avocado seeds showed very high efficiency for Th (IV) removal, achieving 97.3% adsorption from a 750 mg L^−1^ solution at pH 3.0, with a working capacity of 73 mg g^−1^ and 94% uptake within the first five minutes. The process was spontaneous and endothermic, followed pseudo-second-order kinetics and the Freundlich isotherm, and allowed partial regeneration, with 70.5% of adsorbed thorium recovered using 1.0 M nitric acid [125]. While the referenced study demonstrates high Th (IV) removal efficiency, it does not provide an economic assessment, and no specific cost data for avocado seed-derived activated carbon are available; estimates for comparable biomass-derived activated carbons suggest production costs on the order of a few US dollars per kilogram, depending on raw material, activation method, and production scale [126,127].

The above studies revealed that scalability is constrained by material stability, process control, and system integration. Laboratory efficiency represents only one factor in assessing potential industrial readiness, and pilot-scale testing under realistic feed and operational conditions is essential. Furthermore, thorium extraction inevitably generates secondary waste streams, including spent solvents, loaded adsorbents, and acidic sludges containing residual radioactivity. These streams represent both environmental hazards and significant operational costs under NORM regulations [128]. Advanced adsorbents, resins, and MOFs often require complex regeneration procedures, while solvent extraction circuits produce organic waste and acid raffinate streams that necessitate neutralization and stabilization.

Based on the reviewed literature, conventional hydrometallurgical methods, particularly acid leaching combined with solvent extraction, remain the most effective and cost-effective routes for thorium recovery due to their high efficiency, industrial maturity, and predictable operational costs. Emerging technologies such as advanced adsorbents, electrosorption, and biohydrometallurgical approaches offer high selectivity and reduced chemical consumption, but their limited scalability, material stability, and regeneration challenges currently constrain industrial applicability.

These observations indicate that the future of sustainable thorium management could benefit from a shift in research priorities toward integrated assessments. Progress will depend less on marginal gains in laboratory efficiency and more on integrated, holistic assessments from the earliest stages of development. This requires the systematic adoption of LCA to quantify environmental trade-offs and techno-economic analysis (TEA) to reveal true costs, as shown in recent evaluations of monazite processing routes [61,78,129]. For transparency and comparability, future LCA applications could state system boundaries, functional units, allocation procedures for co-produced REEs, electricity mix assumptions, and impact categories [130]. Similarly, TEA should specify the discount rate, plant lifetime, throughput capacity, and treatment of secondary waste management costs [130,131].

**Figure 1:**
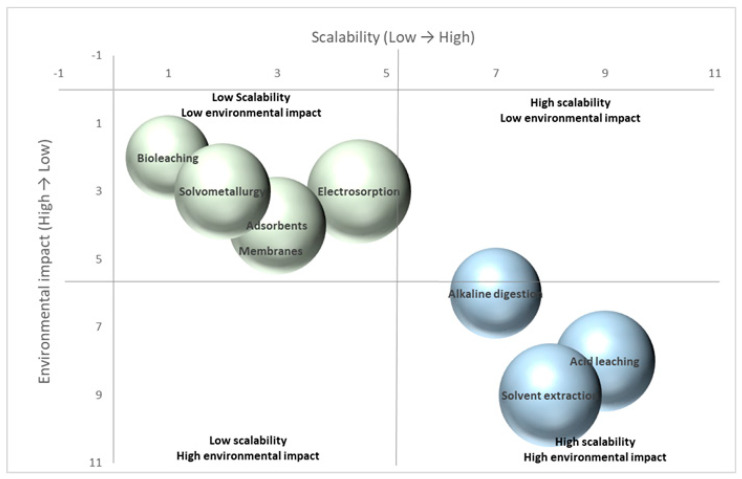
Comparative matrix of thorium extraction and separation technologies by scalability and environmental impact. Blue bubble color—conventional hydrometallurgical processes (acid leaching, alkaline digestion, solvent extraction). Green bubble color—advanced and emerging technologies (adsorbents, electrosorption, membrane processes, biohydrometallurgy, solvometallurgy). Bubble size is proportional to the reported thorium recovery efficiency (%), based on representative values from the literature. Larger bubbles correspond to higher recovery efficiencies. Quadrant boundaries indicate conceptual thresholds between low and high scalability and high and low environmental burden.

To complement the descriptive comparison, Figure 1 synthesizes the relative positioning of conventional and emerging thorium separation technologies in terms of scalability and environmental burden, highlighting the trade-offs between technological maturity and sustainability that are not immediately apparent from tabulated data alone. Notably, no widely investigated thorium separation technology currently occupies the low-scalability/high-impact quadrant, reflecting the tendency for environmentally burdensome processes to persist only when industrially scalable.

Thorium speciation changes throughout extraction, and these transformations directly shape downstream process design. In industrial flowsheets, Th(IV) evolves from lattice-bound forms in minerals to soluble sulfate, nitrate, or chloride complexes during leaching, then to extractant-bound species in solvent extraction, and finally to hydrolyzed or polymeric forms during stripping and neutralization. Each step alters solubility, separability, and waste characteristics [50,132,133,134,135].

For instance, sulfuric acid leaching of monazite generates soluble Th–sulfate complexes at low pH. Extractants such as TBP, D2EHPA, or Cyanex reagents are therefore selected based on their affinity for these dominant aqueous complexes rather than for free Th^4+^, which is rarely present under process conditions [29,136,137]. Downstream, neutralization typically precipitates amorphous Th(OH)_4_, often as fine or colloidal particles [138,139], requiring appropriate solid–liquid separation and stabilization strategies under NORM constraints.

Advanced materials show similar dependencies. Adsorbents and electrosorption systems tested in simplified nitrate or chloride media may perform differently in real leachates containing sulfate, phosphate, carbonate, or organic ligands that modify Th coordination chemistry [110,140,141].

Speciation should therefore be considered a design variable. Integrating equilibrium modeling and phase characterization into flowsheet development can improve extractant selection, control hydrolysis, and anticipate waste forms. Linking speciation analysis with techno-economic and environmental assessment would allow technologies to be evaluated not only by recovery efficiency but also by their ability to generate chemically stable and manageable downstream products.

## 4. Environmental Behavior and Speciation of Thorium

**Figure 2:**
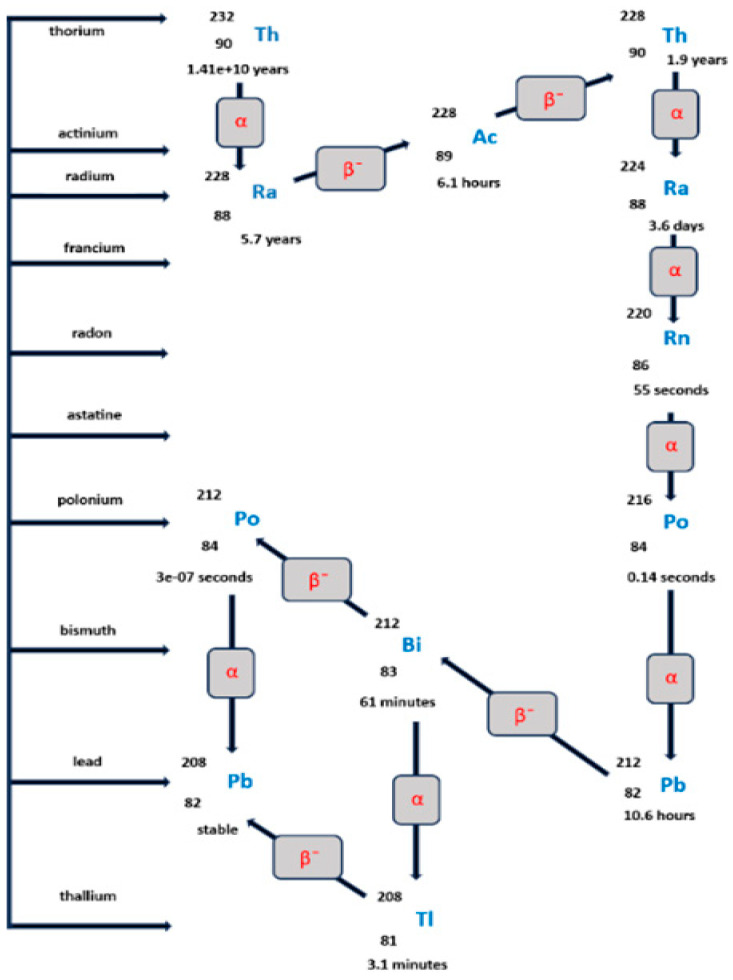
The 4n—thorium disintegration series, initiated by ^232^Th and ending with stable ^208^Pb.

Thorium and some of its decay products are of increasing interest due to their radiological properties and potential applications, such as targeted alpha therapy using radionuclides like ^227^Th, ^229^Th, ^232^Th, ^212^Pb, and ^225^Ac [142,143]. While ^232^Th follows the classical thorium series (4n disintegration chain), ending in stable ^208^Pb (Figure 2), ^227^Th, ^229^Th, and ^225^Ac originate from separate decay chains. Understanding the environmental behavior of thorium is therefore essential for assessing both its long-term radiological persistence and its potential biological impacts.

From a geochemical perspective, thorium occurs predominantly as Th^4+^, a highly charged cation with a large ionic radius, strong Lewis acidity, substantial covalent bonding potential, and a stable + 4 oxidation state. These characteristics are associated with notable interactions with mineral surfaces, organic ligands, and particulate phases, while under certain conditions, Th^4+^ can form soluble complexes and colloidal species. Consequently, thorium mobility is not uniform but depends on local geochemical parameters such as pH, redox potential, mineralogy, ionic strength, and the availability of complexing ligands [25].

In many near-surface environments, thorium exhibits low mobility due to preferential association with solid phases. In karst systems developed on carbonate rocks, thorium binds initially to carbonate surfaces and cements and may be partially released during early weathering stages. However, as dissolution continues, thorium may be retained [144]. Similar retention has been observed in coastal soils and sands of Odisha, India, where thorium is hosted in resistant heavy minerals such as monazite and zircon. Under oxidizing conditions, this mineralogical association tends to limit thorium mobility and may maintain localizedsources of environmental radiation [145]. In the Central Gulf of Gabes, thorium accumulation in surface sediments is associated with phosphogypsum contamination, particularly under acidic conditions with elevated phosphorus and organic matter, promoting localized persistence rather than widespread dispersion [146].

In mineralized regions such as Norway’s Fen Complex, thorium occurs mainly as micrometer-scale inclusions within monazite and niobium-rich minerals, conferring low mobility and limited bioavailability. Resuspension and redistribution may occur under changing hydrological or physical conditions [147]. Comparable patterns have been reported in Lake Bolshoye Yarovoye, where thorium concentrations remain at or below natural background levels, constrained by soil texture, mineral composition, low organic matter content, and salinization [148]. Overall, thorium mobility is generally low but highly site-specific, governed primarily by mineralogical and geochemical context.

Exceptions to thorium immobility are rare under natural conditions but can occur under elevated temperatures or in strongly acidic solutions. In hydrothermal environments, thorium mobility is generally limited. While Th(SO_4_)_2_ complexes can form in sulfate-bearing fluids at moderate to elevated temperatures (175–250 °C), their stability is enhanced at higher temperatures due to positive entropic contributions that can outweigh the endothermic enthalpy of complexation Nevertheless, neutralization of acidic solutions, as often occurs in industrial raffinates, typically precipitates amorphous Th(OH)_4_ nanoparticles, which represent the most common form in natural settings [58,149,150].

Anthropogenic activities further influence thorium speciation and mobility. Mining, chemical processing, and waste management alter thorium’s chemical forms, affecting environmental behavior and exposure potential [59]. During sulfuric acid leaching of monazite, thorium forms neutral sulfate complexes rather than free Th^4+^ ions, increasing solubility and transport potential [72,151]. Subsequent solvent extraction steps, using systems such as Cextrant 230 and Cyanex 923, generate stable organic-phase thorium complexes [83]. Solvent degradation and stripping can remobilize thorium into aqueous raffinates as hydrolyzed or colloidal species, which are difficult to control [122].

Advanced immobilization strategies, including phosphonic acid-grafted metal–organic frameworks (e.g., UiO-66), demonstrate strong inner-sphere thorium complexation and high removal efficiency under laboratory conditions [93]. Nevertheless, the long-term stability of these waste forms is uncertain under variable pH, microbial activity, and ion-exchange conditions. Fine particles and colloids act as critical vectors for post-processing thorium dispersion, particularly in tailings where pH and chloride control is limited [77]. Neutralization of acidic raffinates precipitates amorphous Th(IV) hydroxide [Th(OH)_4_] nanoparticles, which may redissolve under acidic conditions or undergo long-range colloidal transport, further facilitated by wind erosion, seepage, and interactions with natural organic matter [59,152,153].

Speciation strongly influences thorium bioavailability and toxicity. Processes that promote soluble or colloidal species increase biological uptake, while association with resistant mineral phases limits immediate bioavailability yet creates persistent reservoirs capable of remobilization under changing conditions [18]. Experimental studies demonstrate that thorium toxicity depends on the exposure medium and chemical form. In artificial media without bicarbonate, ThO_2_ particles exhibit limited solubility, and toxicity is driven primarily by particle-related interactions rather than dissolved ions [40,154]. Alveolar macrophage studies confirm that insoluble particulate forms are the main driver of biological effects, differentiating thorium from other actinides.

In aquatic assays using OECD TG 201 medium, thorium occurs predominantly as amorphous Th(OH)_4_ precipitates. Toxicity in *Chlorella pyrenoidosa* is mediated by particle attachment, internalization of nanoscale particles, and heteroagglomeration with algal cells, highlighting the importance of chemical speciation and particle behavior over total concentration in predicting biological effects [18,139,155].

Field and geochemical studies reinforce that thorium behavior is controlled by mineralogy, pH, redox conditions, and complexing ligands. In coastal sediments of the Central Gulf of Gabes, phosphogypsum discharge shifts thorium speciation from insoluble hydroxides to dissolved phosphate complexes, increasing mobility and bioavailability [146]. In karst and carbonate environments, thorium released during mineral dissolution is largely retained in residual solids, leading to long-term soil accumulation [144]. In boreal catchments under Th-rich granites, dissolved thorium is primarily transported as organic complexes, emphasizing the role of natural organic matter in enhancing mobility [156].

Overall, thorium toxicity arises from both radiological and chemically mediated mechanisms, including oxidative stress, mitochondrial dysfunction, DNA damage, genomic instability, altered signaling pathways, and inflammation. Effective risk mitigation requires integrating geochemical controls on mobility and speciation with toxicological thresholds and realistic exposure scenarios. High-quality, standardized studies that link contemporary processing conditions to biological outcomes are essential to refine risk assessments, support regulatory frameworks, and guide long-term management strategies for thorium-containing materials.

## 5. Toxicological Effects and Human Health Implications

Current thorium toxicology data are fragmented, with heterogeneous exposure scenarios and limited direct applicability to contemporary industrial conditions. Available evidence is largely derived from historical medical applications, such as Thorotrast use [157,158,159], legacy occupational cohorts in mining and early nuclear industries [17,160,161,162], as well as computational studies [163] or experimental investigations that vary widely in exposure route, dose metrics, and thorium physicochemical form [18,164,165,166]. Consequently, toxicological datasets are often difficult to compare and may limit the robustness of quantitative risk assessments. Inconsistent metrics, heterogeneity in thorium forms, and differences in historical processing conditions complicate quantitative risk assessments [55].

### 5.1. Thorium Exposure, Bioavailability, and Occupational Risk

Occupational exposure to thorium is typically associated with mining, processing, and waste management, with inhalation of fine particles or ingestion of soluble compounds as the main intake pathways. Thorium’s radioactive and chemotoxic nature produces overlapping chemical and radiological effects; acute chemical toxicity typically limits health outcomes before deterministic radiation effects manifest [18,153].

Biokinetic models suggest that lethal internal radiation doses are not expected under modeled exposure scenarios, whereas chemical toxicity sets the upper bound for acute risk [167]. Epidemiological studies show measurable systemic retention and associated health effects. Indian monazite-processing workers experienced low but chronic thorium intake (~2 µg/day via food; ~0.02 µg/day via inhalation), with accumulation in lungs, bones, and muscles and elevated rates of cardiovascular disease, sterility, and genetic disorders [168,169]. Western Australian mineral sands workers displayed committed internal doses exceeding 20 mSv/year due to low solubility and slow pulmonary clearance [160,167]. Conversely, dietary intakes in German volunteers (~10 ppm thorium) did not significantly increase excretion, indicating low gastrointestinal absorption [170].

Experimental evidence has reported higher toxicity of insoluble thorium compounds, such as thorium dioxide (ThO_2_), compared to soluble forms [171]. Thorium-based targeted radionuclide therapies (^227^Th conjugates) elicit dose-dependent DNA damage, oxidative stress, and immunogenic cell death, showing clinical safety and antitumor activity [172,173,174].

Therefore, chemical toxicity dominates acute outcomes, while long-term retention poses chronic exposure concerns.

### 5.2. Molecular Mechanisms of Thorium Toxicity

Chronic low-level thorium exposure induces systemic effects, including hematological changes, neurochemical alterations, and oxidative stress [175].

^232^Thorium’s alpha emissions can result in localized cellular damage, particularly following inhalation of thorium dust. In WI26 lung epithelial cells, ThO_2_ caused dose- and time-dependent cytotoxicity, ROS generation, lipid peroxidation, mitochondrial dysfunction, DNA double-strand breaks, and activation of HSP90 and ATM signaling pathways [176]. Thorium exposure disrupts Complex IV of the mitochondrial electron transport chain, reducing ATP synthesis and enhancing ROS leakage [177]. Thorium exposure also activates NF-κB, IL-6, and TNF-α in vitro and in vivo, amplifying ROS production and promoting pro-tumorigenic microenvironments [171,178,179,180]. In this sense, it has been proven that chronic exposure dysregulates Wnt/β-catenin signaling, driving hepatic carcinogenesis through transcriptomic and proteomic alterations in mice [181].

DNA damage from high-LET alpha particles is highly clustered, persistent, and repaired inefficiently, leading to chromosomal aberrations and genomic instability [182,183,184,185].

Persistent DNA damage and oxidative stress activate stress-response pathways including ATM/ATR and p53, shifting cellular outcomes from transient repair to senescence or apoptosis [186,187].

Epigenetic modifications observed under high-LET radiation, including DNA methylation and histone modification changes, likely contribute to long-term genomic instability [188,189].

Thorium, primarily as Th(IV), exhibits strong and selective interactions with biologically relevant organic molecules, particularly carboxylate-containing amino acids, phosphorylated peptides, and proteins, forming stable complexes that influence its mobility, bioavailability, and long-term behavior in environmental and biological systems [190]. Molecular dynamics and spectroscopic studies demonstrate that Th(IV) preferentially binds to acidic residues (aspartic acid, glutamic acid), phosphorylated motifs, and carboxylates on protein rings, as seen in hyperphosphorylated proteins such as osteopontin and in ferritin, where up to 2840 Th atoms per protein can be accommodated at physiological pH without involving the ferrihydric core, with potential contributions from carbonate ions [191,192,193]. Experimental evidence from peptide-based models confirms that Th(IV) forms highly stable complexes, such as tetra-phosphorylated peptide Th_2_(pS1368) and ternary Th_2_(pS16) (pS1368) structures, which can be detected at sub-nanomolar levels with nanopore sensors, even in the presence of competing metal ions [194]. Computational studies, including DFT and molecular dynamics parameterized for Th^4+^, reproduce these binding geometries and provide quantitative insights into coordination modes, energetics, and pH-dependent stability [195]. Collectively, these findings support the use of Th(IV) as a model actinide for investigating actinide–biomolecule interactions, informing nuclear toxicology, environmental monitoring, and strategies for safe management and decorporation of actinide contaminants [190].

Such molecular-scale behavior is essential for understanding long-term biological retention and exposure, but does not directly address technological valorization or process-level trade-offs.

**Figure 3:**
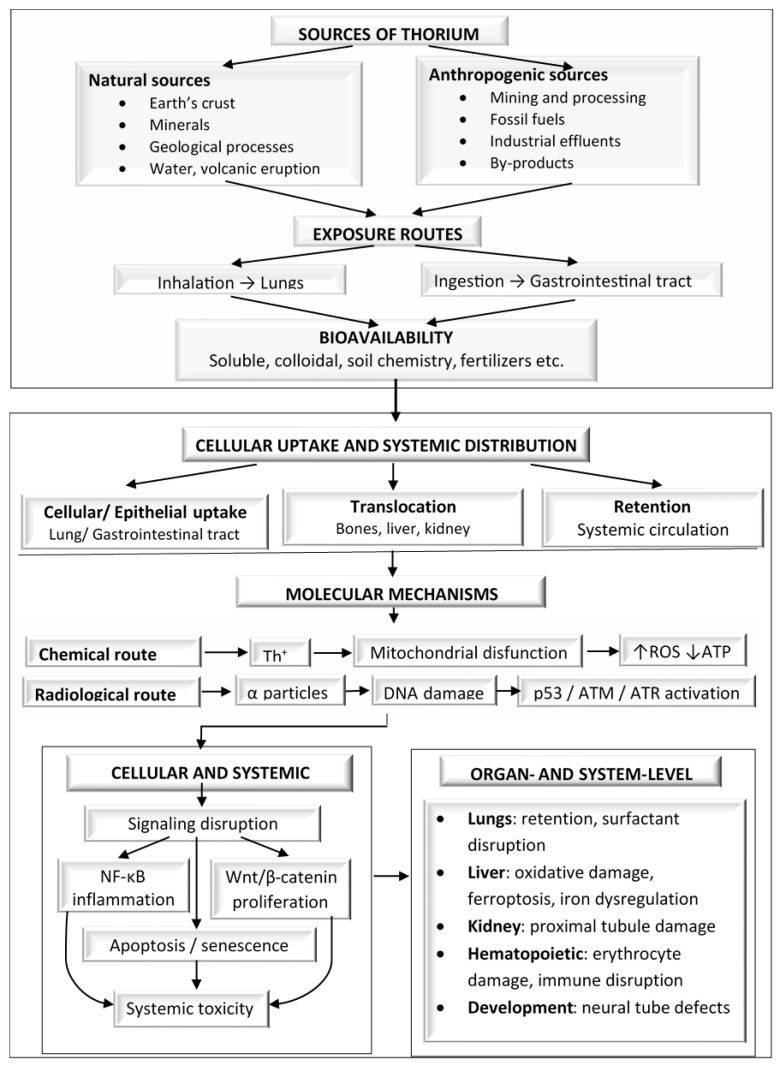
Integrated schematic overview of thorium exposure pathways, cellular toxicity mechanisms, systemic effects, and therapeutic implications.

Figure 3 summarizes the main sources and exposure routes of thorium, the key modifiers of its bioavailability, the downstream molecular and cellular toxicity mechanisms, and the resulting organ-level effects.

## 6. Integrating Valorization and Risk: Technological Trade-Offs and Knowledge Gaps

Assessment of thorium valorization may benefit from considering the extraction yield or separation efficiency. Laboratory-scale studies often report high performance but may not reflect the complexities of industrial implementation, where environmental, occupational, and economic considerations become increasingly relevant. A comprehensive approach requires integrating technological performance with environmental risk, economic feasibility, and long-term system behavior.

LCA highlights these trade-offs. The LCA boundary is typically defined as cradle-to-gate or gate-to-gate, with 1 kg of recovered thorium (or equivalent thorium compound) as the functional unit. Impact categories typically include global warming potential (kg CO_2_-eq), cumulative energy demand (MJ/kg), acidification potential, and human toxicity indicators. Assumptions regarding electricity mix, reagent regeneration rates, and waste neutralization significantly influenced reported outcomes [78,196]. Comparative LCAs in Malaysia show that conventional solvent extraction from monazite is dominated by energy- and reagent-intensive steps, with thorium sulfate production contributing approximately 45 kg CO_2_-eq per kg Th. Electrosorption-based recovery from radioactive residues requires roughly one-third of the energy input and exhibits lower toxicity and emission profiles [78]. Nonetheless, uncertainties remain regarding electrode lifetime, performance under variable electricity mixes and climatic conditions, and the absence of reliable industrial-scale cost data. A parallel LCA comparing activated carbon and graphene-based electrodes illustrates that material choice significantly affects both recovery efficiency and environmental burden while leaving open questions about scalability and lifecycle emissions [197].

TEA studies are typically based on discounted cash flow models, incorporating capital expenditure, operational expenditure, reagent consumption, energy demand, labor, and waste treatment costs. Key financial indicators include net present value, internal rate of return, return on investment, and discounted payback period, often evaluated under specific assumptions regarding plant capacity, feed composition, and thorium or rare earth market prices [74,198,199]. TEA emphasizes scale-dependent trade-offs. Large batch sizes reduce unit production costs but require high capital investments, constraining return on investment and increasing financial risk [40,74]. Sensitivity analyses frequently identify raw material cost, reaction duration, and equipment investment as critical variables, underscoring persistent knowledge gaps in process integration and commercial feasibility beyond pilot scale. Integrating thorium recovery with rare earth element processing can improve economic viability and mitigate waste-related risks, but favorable outcomes depend on market conditions and careful cost allocation [61]. This suggests potential sensitivity to commodity price fluctuations, which is rarely addressed in laboratory-driven research.

Advanced separation materials illustrate additional technological trade-offs. Acid-stable frameworks, functionalized carbons, resins, and green solvent systems can achieve near-quantitative Th(IV) capture under highly acidic or radioactive conditions [31,43,77,200,201]. These materials simultaneously support resource recovery and risk mitigation, yet they introduce new uncertainties related to synthesis complexity, regeneration efficiency, secondary waste generation, radiolytic stability, and regulatory classification of spent materials. Often, environmental and occupational risks are shifted rather than fully eliminated.

From a lifecycle perspective, additional uncertainties arise from radiolytic stability and material durability. Alpha-emitting decay products associated with ^232^Th may accelerate structural degradation of functionalized adsorbents, membranes, or organic solvents, increasing replacement frequency and consequently the embodied energy and carbon footprint per kilogram of recovered thorium [123,202,203]. Although several zirconium-based MOFs (e.g., UiO-type structures) demonstrate acid resistance, systematic data on long-term structural integrity under continuous exposure to alpha-emitting Th(IV) species are scarce. The potential radiolytic degradation of functional groups may compromise selectivity and regeneration efficiency over extended operational cycles [204,205]. Moreover, the synthesis of advanced materials such as MOFs, graphene derivatives, or deep eutectic solvents may involve multi-step procedures with significant energy and reagent demand [206,207]. In several reported systems, the environmental burden of material production may equal or exceed that of the separation step itself [208,209]. These aspects are rarely incorporated into existing LCAs, which are often based on model solutions rather than radioactive, compositionally complex process streams.

Reactor-focused analyses further highlight the gap between technological promise and environmental integration. Economic evaluations of thorium-compatible molten salt reactors indicate favorable financial performance and inherent safety advantages, yet chemical speciation, waste forms, and long-term environmental behavior are seldom considered across the full fuel cycle [204,210]. Financial modeling using discounted cash flow and Monte Carlo simulations in Bangka Belitung, Indonesia, revealed strong economic indicators (NPV USD 338.7 million; IRR 13.06%; profitability index 1.40; discounted payback 11.19 years) but also exposed potential downside risks from construction delays, public acceptance, fuel supply continuity, regulatory shifts, and efficiency reductions, emphasizing the importance of integrate economic valorization with environmental and safety [199].

Across studies, a recurring trend has been observed: high extraction efficiency is often pursued in isolation, with limited attention to downstream consequences such as secondary waste stabilization, occupational exposure, decommissioning, and long-term monitoring. Regulatory frameworks (IAEA Safety Standards, Euratom Treaty) establish requirements for dose control and environmental monitoring, yet documented contamination cases reveal persistent gaps in integrating thorium resource development with effective environmental governance [64,115,211,212].

Table 2 provides an integrated summary of the performance and limitations of these processes, reinforcing the need for holistic assessments encompassing techno-economic feasibility, environmental impact, and operational safety.

Emerging technologies are promoted as cleaner or more selective alternatives but bring hidden constraints, including complex synthesis, narrow operational windows, and uncertain long-term stability. Functionalized adsorbents and membrane-based systems may reduce solvent use but introduce new challenges related to material regeneration, waste classification, and process scalability. In many cases, these trade-offs shift rather than eliminate environmental and occupational risks.

From a techno-economic standpoint, the cost structure of emerging thorium separation materials remains insufficiently characterized. Laboratory demonstrations rarely include full capital expenditure for material synthesis, scale-up of functionalized sorbents, or replacement rates under radiological exposure [203,213]. In thorium-bearing systems classified as NORM, additional operational costs arise from radiological monitoring, shielding, waste stabilization, and long-term liability management [30,214]. When regeneration efficiency declines below laboratory-reported values, the effective cost per kilogram of recovered thorium may increase substantially. These factors are seldom incorporated into published techno-economic assessments, which tend to extrapolate from short-term batch experiments rather than continuous, radiation-exposed industrial operation.

Several knowledge gaps continue to limit a comprehensive integrated assessment. First, standardized metrics for comparing technologies across efficiency, risk, and cost are lacking. Second, economic analyses often exclude waste management, decommissioning, and long-term monitoring costs, leading to overly optimistic feasibility assessments. Third, the interaction between chemical speciation, environmental mobility, and human exposure remains insufficiently quantified under realistic industrial scenarios. Addressing these gaps requires a shift from laboratory-centric optimization toward lifecycle-informed process design that minimizes secondary waste, reduces occupational exposure, and aligns with regulatory and environmental constraints. Such an integrated approach could facilitate the transition of thorium valorization from conceptual development to responsible industrial implementation.

**Table 2:** Thorium recovery methods: performance, scalability, and key limitations.

Process/Material	Type of Process	Key Performance	Scalability & Limitations	References
Activated carbon (avocado seeds)	Adsorbent	97.3% Th(IV) removal; 73 mg·g^−1^ capacity; 94% uptake in 5 min	Partial regeneration (70.5%), lab-scale; industrial scaling requires optimization	[125]
MOFs (SZ-7 zirconium phosphonate)	Adsorbent	Near-quantitative Th(IV) capture from acidic wastewater (pH < 2)	Exceptional acid stability; regeneration and long-term scaling unknown	[77]
Polyethylenimine-functionalized graphene oxide	Adsorbent	38.17 mg·g^−1^ capacity; SF > 100	High selectivity and recyclability; industrial deployment limited by synthesis and scale	[215]
Polycinnamic acid resin	Adsorbent	Selective Th(IV) removal from REE solutions	Requires precise polymer synthesis; large-scale production uncertain	[87]
TOPO/XAD-7 resin	Resin/Solid-phase extractor	59.79 mg·g^−1^ capacity; >99.5% recovery in dynamic columns	High acid and radiation tolerance; scale-up requires consideration of competing ions	[216]
N,O-donor hybrid heterocyclic extractants	Solvent/Extractant	>90% Th(IV) recovery; SF 50–110	Effective under controlled HNO_3_; multi-step stripping needed; scale-up challenges	[217]
Deep eutectic solvents (2-hexyldecanoic acid-based)	Solvent/Green extractant	>98% Th extraction; SF > 1000 vs. REEs	Lab-scale; long-term stability, radiolytic resistance, and industrial scalability unresolved	[200]
HCl multi-step leaching (Longnan, China)	Acid leaching	~99% Th leaching; >98% REE recovery	Reduces acid consumption; secondary waste management and extractant sustainability need evaluation	[43]
Alkaline fusion (ThO_2_ from monazite)	Alkaline/Conventional process	ROI 21.92% at 1 t batch; payback 4.56 years	High capital for large batch; sensitive to raw material cost and reaction time	[74]
Monazite processing (Korea, eco-friendly)	Conventional/Integrated REE-Th-U	Th and U recovery with REEs; payback ~4.5 y	Operational cost dominated by materials/reagents; market-dependent	[61]

While ^232^Th has been proposed as an alternative nuclear fuel through its conversion to ^233^U, the fuel system involves radiological and operational challenges. ^233^U is highly radioactive with alpha emissions that require appropriate shielding and controlled handling protocols throughout fuel fabrication, irradiation, and reprocessing. Long-lived decay products contribute to waste management complexity, necessitating robust containment, storage, and monitoring strategies over extended timescales. Moreover, variations in thorium feedstock composition and chemical form influence neutron flux behavior and reactor kinetics, creating operational uncertainties. Consequently, any valorization of thorium resources for energy purposes must integrate these radiological and engineering considerations into lifecycle assessments and risk management frameworks, alongside environmental and occupational safety evaluations already discussed for extraction and separation processes [137,218].

## 7. Future Research Directions and a Framework for Sustainable Thorium Management

The future of thorium utilization may benefit from a holistic approach that balances technological performance with regulatory, environmental, and occupational safety imperatives. Incremental gains in laboratory-scale separation efficiency may be insufficient if they do not translate into processes that are scalable, safe, and environmentally responsible. A recurring gap exists between promising lab results and industrial implementation, often due to underestimation of non-technical barriers, including heterogeneous regulations for NORM, undefined waste classification for novel streams, and limited predictive models for occupational exposure [25,36,216].

Future research should adopt an integrated, decision-oriented paradigm that prioritizes technologies capable of simultaneously minimizing secondary waste, simplifying regulatory compliance, and enhancing intrinsic safety. High-performance adsorbents such as functionalized COFs or activated carbons should be evaluated not only for chemical selectivity but also for stability in complex feedstocks, recyclability, and the environmental footprint of synthesis and disposal [86,201,203]. For thorium-specific applications, this evaluation should explicitly include radiolytic resistance, long-term regeneration under alpha-emitting conditions, lifecycle emissions associated with material synthesis, and full cost accounting, including NORM compliance and end-of-life disposal. Standardized benchmarking protocols would facilitate more consistent evaluation of recovery efficiency, reagent consumption, waste generation, energy intensity, and scalability on a common basis.

**Figure 4:**
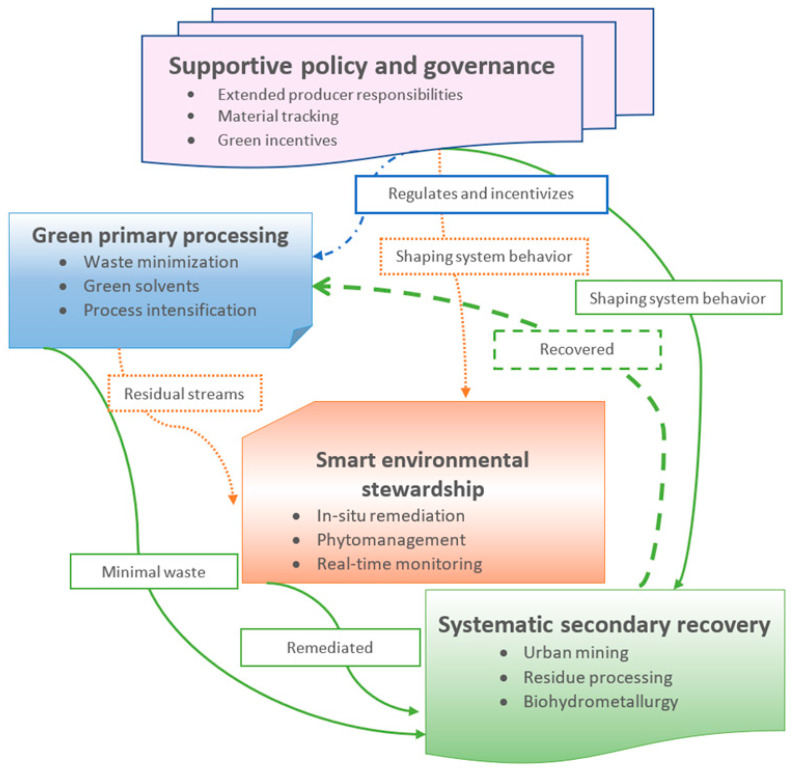
A sustainable circular economy framework for thorium management.

A sustainable thorium management framework can be conceptualized as a circular system integrating four pillars, as illustrated in Figure 4:

1. Green Primary Processing: Minimization of waste at source through process intensification, green solvents, and membrane-assisted extraction technologies [16,142,219].
2. Systematic Secondary Recovery: Reclamation of thorium from industrial residues, end-of-life products, and other secondary streams using advanced hydrometallurgical and bio-hydrometallurgical approaches [63,101,220].
3. Smart Environmental Stewardship: Site-specific remediation through in situ immobilization, phytomanagement, and real-time monitoring to ensure minimal ecological impact [12,95,221].
4. Supportive Policy and Governance: Regulatory frameworks, extended producer responsibilities, material tracking, and green incentives that guide and facilitate the circular management of thorium [222].

The path forward for thorium is systemic rather than reliant on isolated breakthroughs. Advancement may be facilitated by interdisciplinary approaches, standardized evaluation protocols, and consideration of circular economy principles, ensuring that thorium management becomes both environmentally sustainable and industrially feasible.

## 8. Conclusions

This review critically examined thorium valorization by integrating extraction technologies with environmental behavior, toxicological evidence, and regulatory constraints. Several robust conclusions emerge from the current body of literature.

First, thorium cannot be effectively managed as an isolated resource. Its persistent association with rare earth element production, mining residues, and industrial by-products means that thorium recovery, waste handling, and exposure control are inherently interconnected. Conventional hydrometallurgical routes are currently reported as technologically mature and infrastructure-compatible options. However, their deployment is inseparable from significant challenges related to high reagent consumption, secondary waste generation, and long-term environmental and occupational liabilities.

Methods reported to date that combine high chemical efficiency with established industrial scalability have been highlighted as particularly relevant for thorium separation. At present, conventional acid leaching followed by solvent extraction remains the benchmark for thorium recovery, whereas advanced adsorbents, electrosorption, and bio-based methods offer valuable insights into selective and environmentally safer recovery, but require further development and techno-economic evaluation before large-scale deployment.

Second, laboratory-scale performance metrics may not directly reflect potential performance under industrial conditions. High extraction efficiencies and selectivities achieved under controlled conditions do not necessarily translate into scalable, regulatory-compliant, or sustainable processes. Many advanced and emerging technologies reduce solvent use or improve selectivity, but often shift environmental and occupational risks rather than eliminating them. Economic feasibility, long-term material stability, and regulatory classification of secondary waste streams remain insufficiently addressed in much experimental research.

Third, assumptions regarding thorium’s environmental immobility and predictable toxicological behavior are not consistently supported when chemical speciation, process-induced transformations, and exposure pathways are considered. Occupational risk is primarily governed by inhalation and ingestion of bioavailable thorium forms, where chemical toxicity dominates acute effects and long-term retention contributes to chronic exposure. At the mechanistic level, thorium toxicity is mediated by oxidative stress, alpha-induced DNA damage, and strong biomolecular interactions, leading to cumulative cellular, organ-level, and systemic effects.

Experimental and field studies reported thorium toxicity across aquatic, mammalian, and developmental models. Industrial and energy-related activities amplify thorium dispersion and persistence in the environment. While mineralogical sequestration can limit short-term mobility, it complicates long-term remediation and monitoring. These findings underscore the necessity of integrating mineralogical, geochemical, and toxicological knowledge into comprehensive risk assessment and mitigation strategies.

Overall, thorium valorization is defined by interlinked trade-offs between recovery efficiency, environmental and occupational risk, capital intensity, and long-term system stability. Persistent knowledge gaps remain in scaling behavior, lifecycle emissions, waste form durability, and the coupling between chemical speciation and realistic exposure scenarios. Addressing these gaps requires a shift away from laboratory-centric optimization toward lifecycle-informed, system-level process design aligned with regulatory and environmental constraints.

Finally, sustainable thorium management depends on the coordinated integration of five critical conditions: (i) pilot-scale validation to confirm performance under realistic feedstocks and operational conditions; (ii) LCA to quantify environmental trade-offs, including secondary waste, solvent use, and radiological hazards; (iii) techno-economic analysis to assess capital and operational costs, payback periods, and market sensitivity; (iv) demonstrated robustness and regenerability of adsorbents, resins, and framework materials under industrial conditions; and (v) integrated risk management encompassing worker safety, compliance with NORM regulations, and effective effluent detoxification. Only through such a comprehensive and balanced framework can thorium technologies progress from conceptual promise to responsible and sustainable industrial implementation.

## Figures and Tables

**Table 1:** Technical overview, advantages, limitations, and development stage of thorium separation techniques in rare earth element processing.

Separation Method for Thorium	Key Technical Advantages	Major Limitations/Challenges	Technology Maturity and Key Evidence	References
Acid leaching (H_2_SO_4_, HCl, HNO_3_)	High thorium dissolution efficiency from monazite; well-integrated into conventional rare earth processing flowsheets.	Generation of large volumes of acidic, radioactive waste; high reagent consumption and corrosion issues.	Commercial process. Subject of recent techno-economic evaluations for integrated industrial flowsheets	[61,72,79]
Alkaline leaching/fusion (NaOH, KOH)	Provides improved control over thorium separation; can reduce co-dissolution of rare earths compared to some acid routes.	High energy input required; generates alkaline waste that needs neutralization.	Commercial process (conditional). Economically evaluated for ThO_2_ production.	[74,75]
Solvent extraction (TBP, D2EHPA, etc.)	High selectivity and efficiency for Th/RE separation; proven in scalable, multi-stage contactor systems (e.g., mixer-settlers).	Generates organic secondary waste; risks of solvent degradation and crud formation; requires rigorous safety protocols.	Commercial unit operation. Solvent extraction circuits are designed and optimized for “nuclear industry applications”.	[71,103,122]
Ion-exchange resins	Highly effective for polishing and recovering thorium from dilute or low-concentration streams (e.g., wastewater).	Limited kinetic rates and adsorption capacity; generates secondary waste from resin regeneration.	Pilot-scale/auxiliary. Primarily demonstrated at laboratory scale for separation from complex sulfate media.	[103]
Advanced solid adsorbents—MOFs, COFs, functional polymers	Exceptional selectivity and high adsorption capacity under controlled laboratory conditions.	Complex and costly synthesis; performance often degrades in complex, real matrices; challenges with material stability and recycling.	Fundamental research. Novel materials described for “selective extraction” in batch lab studies.	[93,95,106,107]
Membrane-based processes	Potential for continuous, low-chemical-input operation with a small footprint.	Susceptibility to fouling and scaling; low throughput; limited long-term stability data for radioactive feeds.	Emerging technology (pilot-concept). Systems like “continuous-flow electrosorption” are “advancing towards technology readiness”.	[85,123]
Bio-hydrometallurgy (bioleaching, biosorption)	Low energy and chemical consumption in principle; potential for processing low-grade or secondary resources.	Very slow kinetics; sensitive to environmental conditions; poor reproducibility and control at larger scales.	Early-stage research. Reviewed as a “sustainable approach” with focus on “future prospects” rather than current application.	[99,101,121]
Electrosorption/electrochemical recovery	Electric-field-assisted adsorption enhances thorium uptake, selectivity, and reusability; reduces chemical use; compatible with renewable energy.	Limited electrode stability and availability; low throughput; sensitive to competing ions; lab-scale demonstration	Emerging technology. High lab-scale recovery (>3000 mg·g^−1^ GF-AO; ~124 mg·g^−1^ g-C3N4); industrial scale-up not yet validated	[31,90,110]

Note: Technology maturity reflects the highest demonstrated readiness level reported in the cited literature. Reported advantages and limitations are method-specific and may vary with feed composition, operating conditions, and process integration. References correspond to representative studies and reviews rather than an exhaustive survey.

## Data Availability

No new data were created or analyzed in this study. Data sharing is not applicable to this article.

## Abbreviations

The following abbreviations are used in this manuscript:

ACE	Activated carbon electrodes
COFs	Covalent organic frameworks
DMHMP	Di(1-methyl-heptyl) methyl phosphonate
GBE	Graphene-based electrodes
g-C3N4	Graphitic carbon nitride
GF-AO	Amidoxime-modified graphite felt
H_2_SO_4_	Sulfuric acid
HCl	Hydrochloric acid
HNO_3_	Nitric acid
LCA	Life cycle assessment
MOFs	Metal–organic frameworks
NORM	Naturally occurring radioactive materials
REE	Rare earth element
TBP	Tributyl phosphate
TEA	Techno-economic analysis
Th	Thorium
